# Cry-based infant pathology classification using GMMs

**DOI:** 10.1016/j.specom.2015.12.001

**Published:** 2016-03

**Authors:** Hesam Farsaie Alaie, Lina Abou-Abbas, Chakib Tadj

**Affiliations:** MMS Lab, Department of Electrical Engineering, École de Technologie Supérieure, Université du Québec, 1100 rue Notre-Dame Ouest, Montréal, QC, Canada, H3C 1K3

**Keywords:** Gaussian mixture model, Universal background model, Mel-frequency Cepstral Coefficient, Likelihood ratio scores, Newborn infant cries, Expiratory and inspiratory cry

## Abstract

Traditional studies of infant cry signals focus more on non-pathology-based classification of infants. In this paper, we introduce a noninvasive health care system that performs acoustic analysis of unclean noisy infant cry signals to extract and measure certain cry characteristics quantitatively and classify healthy and sick newborn infants according to only their cries. In the conduct of this newborn cry-based diagnostic system, the dynamic MFCC features along with static Mel-Frequency Cepstral Coefficients (MFCCs) are selected and extracted for both expiratory and inspiratory cry vocalizations to produce a discriminative and informative feature vector. Next, we create a unique cry pattern for each cry vocalization type and pathological condition by introducing a novel idea using the Boosting Mixture Learning (BML) method to derive either healthy or pathology subclass models separately from the Gaussian Mixture Model-Universal Background Model (GMM-UBM). Our newborn cry-based diagnostic system (NCDS) has a hierarchical scheme that is a treelike combination of individual classifiers. Moreover, a score-level fusion of the proposed expiratory and inspiratory cry-based subsystems is performed to make a more reliable decision. The experimental results indicate that the adapted BML method has lower error rates than the Bayesian approach or the maximum a posteriori probability (MAP) adaptation approach when considered as a reference method.

## Highlights

• We characterize the distributions of the acoustic features of infant cry signals with GMMs as a universal background model.
• An adapted BML method is presented to derive either healthy or pathology subclass models from the GMM-UBM.
• A score level fusion of obtained log-likelihood ratio scores from both expiratory and inspiratory sounds is performed.
• The proposed cry-based diagnostic system is used to classify healthy and sick infants.
• Subjective results indicated that the proposed method can perform better than the Bayesian adaptation.

## 1 Introduction

Crying is the first clear sign of life that is observed shortly after a baby's live birth. Although there have been some books and products that were created through the years to unlock the secret language of babies, their potential for use in the early diagnosis and treatment of newborns remains largely in an open and undeveloped state. The results of these studies highlight the existence of some cry attributes in sick infants that are rarely observed in the cries of healthy infants (Wasz-Hockert et al., 1985, Wasz-Hockert et al., 1968, Benson and Haith, 2010). Instead, these attributes occur frequently in the cries of sick infants who suffer from different medical diseases and conditions. Therefore, infant cry characteristics reflect the integrity of the central nervous system.

### 1.1 Early studies and birth defects

Many early researchers defined several cry characteristics and presented their common values, such as the fundamental frequency, formants, cry modes, cry latency, phonation, hyperphonation, and dysphonation (Wasz-Hockert et al., 1985, LaGasse et al., 2005, Lederman, 2002, Newman, 1985). Gradually, detailed acoustic analysis, which measures and compares the acoustical characteristics of newborn infant cry signals, shows hidden diagnostic potential of cry signals for the basic cry types and the cries of infants in pathological conditions such as brain damage, central nervous system diseases and Down's syndrome (Wasz-Hockert et al., 1985, Wasz-Hockert et al., 1968, Michelsson, 1971, Michelsson and Michelsson, 1999, Partanen et al., 1967). It appears that some of the symptoms are not always recognized or even do not appear for months or years; thus, it might be too late for treatment after clinical symptoms start, especially in countries that do not have well-established health services.

**Fig. 1:**
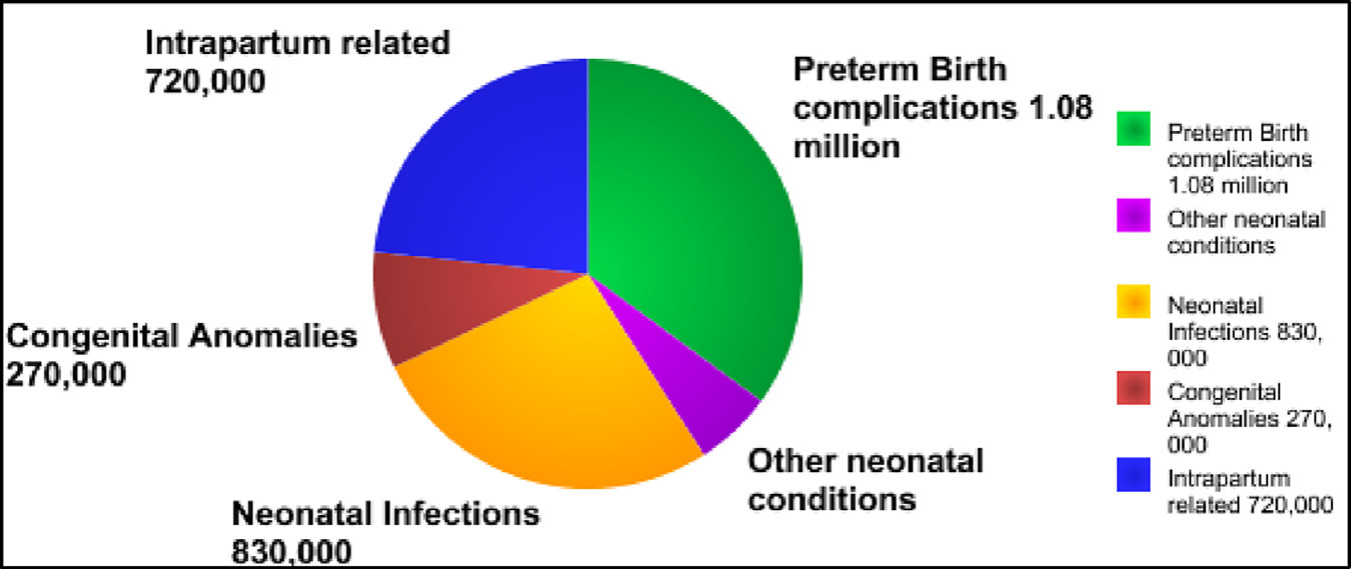
Leading causes of infant deaths in 193 countries in 2010.

As Fig. 1 shows, congenital anomalies and preterm births were the dominant causes of approximately 2.7 million infants deaths in 193 countries in 2010 (Congenital anomalies, 2014). Statistical reports by the World Health Organization (WHO) (Congenital anomalies, 2014) and Center for Disease Control and prevention (CDC) (Anon, 2008) state that congenital anomalies or birth defects affect approximately 1 in 33 infants born every year. Moreover, in spite of the fact that the U.S. and Canada are highly developed countries, the results of an investigation of early infant mortality rates in 176 countries indicate that the U.S. and Canada had the 1st and 2nd worst rates in the developed world, respectively, by 2.6 and 2.4 first-day deaths per 1000 births (SaveTheChildren, 2013). It is worthwhile mentioning that approximately 1 percent of the world's infant deaths occur in developed countries, and the situation is worse for many developing countries. However, it is easier to identify a baby who has structural problems such as cleft lip; on the other hand, symptoms of some defects might be invisible and hidden from sight. Therefore, we believe that by providing an inexpensive health care system that does not have complex and advanced technology for poor mothers with newborn babies in low-income countries, more babies can survive beyond the first months of life.

There are a substantial number of maternal and environmental issues that can raise the risks of several complications and associated anomalies, such as the gestational age, birth weight, consanguinity, maternal age, multiple gestations, maternal infection during pregnancy, socioeconomic factors and maternal nutritional status. For example, the gestational age is a noteworthy predictor of infant health conditions within the normal range of 37–41 weeks for babies who are fully developed (full term). Premature birth, even only a few weeks early, increases the chance of birth defects or infant death in such a way that in the U.S., the 2010 mortality rate for very early preterm (under 32 weeks) births was 74 times worse than that of full-term infants (Mathews and MacDorman, 2013).

These official statistics, which is completely independent of the information in the infant cries, can provide more information about the chance that an infant is born with a specific congenital disease. Moreover, there are other independent sources of information that are related to the physiological condition of the newborn infants that can be useful in a similar way in multimodal biometric systems, which use multiple independent sources of information and indeed provide a more reliable system. However, in this paper, we are curious about examining only the ability of information that is embedded in infant cries to differentiate between several pathologies in spite of the other sources.

This approach encouraged us to be ambitious and develop a newborn cry-based diagnostic system for the care of birth defects after birth by identifying some possible physiological disorders and birth defects. This early intervention can definitely save the lives of many infants and protect them from some physical, intellectual, visual or auditory impairment before severe disabilities might be caused.

### 1.2 Related studies

The leading role in the classification of the infant cry is how to scientifically discriminate between different neonatal health statuses, only on the basis of their cry signals besides the health examination of infants and other predictors of child health. In recent years, several machine learning and classification algorithms, such as artificial neural networks (Orozco and Garcia, 2003, Cano et al., 2006, Hariharan et al., 2011), radial basis function (RBF) networks (Cano Ortiz et al., 2004), support vector machines (SVMs) (Amaro-Camargo and Reyes-García, 2007), Naïve Bayes (Amaro-Camargo and Reyes-García, 2007), Genetic Selection of a Fuzzy Model (GSFM) (Rosales-Pérez et al., 2012) have demonstrated the ability to recognize cry patterns and make intelligent decisions based on the available training databases. According to our knowledge, case subjects of binary classification tasks in most of the previous works (Orozco and Garcia, 2003, Cano et al., 2006, Hariharan et al., 2011, Cano Ortiz et al., 2004, Amaro-Camargo and Reyes-García, 2007) were normal infants and those infants suffering from hypo acoustic (deaf) or asphyxia. On the contrary, the principal aim of the proposed system is to broaden the diagnostic system to address the most life-threatening illnesses and defects that occur in newborn infants in the early days of their life. It is worthwhile mentioning that it is sometimes not easy to collect a large number of samples to represent a general cry pattern. The failure to achieve the lowest possible error rate is the main drawback of having no acceptable cry database. For this reason, learning from a small, incomplete set of samples is of practical interest.

Finite mixture models are a flexible and powerful probabilistic tool for modeling univariate and multivariate data to perform modeling and classification tasks (McLachlan and Peel, 2004). In this paper, we employ the Gaussian mixture model (GMM), which is a powerful model for representing almost any distribution. The Gaussian mixture model is computationally inexpensive and is based on a well-understood statistical model that can be viewed as a hybrid between a parametric and nonparametric density model. Moreover, there are many advantages that are claimed when using GMM as the likelihood function (Reynolds et al., 2000). The expectation-maximization (EM) algorithm is a common method for maximum likelihood learning of finite GMMs; this approach has some advantages over other learning methods, such as a gradient-based approach (Xu and Jordan, 1996).

In our previous studies (Farsaie Alaie and Tadj, 2012, Alaie and Tadj, 2013), we have introduced a working prototype to train a GMM in an incremental and recursive manner; this method is called the adapted boosting mixture learning Method (BML). The proposed method trains finite mixture models by a pool of Gaussian components. Partial and global updating methods are used in model parameter estimation processes to speed up the learning process and converge to a more robust and reliable estimation of a new mixture component. The selected strategy to stop the adding process is a criterion-based approach called Bayesian Information Criterion (BIC). We have shown that the proposed method has better performance than the traditional EM-based re-estimation algorithm as a reference system for the classification of the infants’ cries. In a binary classification task, the system discriminated a test infant's cry signal into one of two classes, namely, healthy infants and sick newborn babies with selected diseases.

In this paper, we describe the development and evaluation of a Gaussian Mixture Model-Universal Background Model (GMM-UBM) system that is applied to an infant cry expiration and inspiration corpora for the enrolled health conditions. This newborn cry-based diagnostic system can be referred to as the GMM-UBM health-condition verification/detection system.

The remainder of this paper is organized as follows. In Section 2, we present recording procedure and our cry database. Next, in Section 3, the feature extraction procedure is explained. Section 4 presents our classification approach, and Section 5 describes experiments and the results of each health-condition detector using our cry database. Finally, conclusions and future directions are presented in Section 6.

## 2 Recording procedure and cry database

The recordings were made in the neonatology departments of several hospitals in Canada and Lebanon. We performed the recording process by converting the analog cry signal to an uncompressed digital audio format that is suitable for storing an original recording in a wav file. Each infant's cry was recorded by an Olympus hand-held digital 2-channel recorder, placed 10–30 cm away from the infant's mouth, at a sampling frequency of 44.1 kHz and a sample resolution of 16 bits. A neonatal intensive-care unit (NICU) is a special system of care for newborn infants who are sick or premature or generally need more medical attention due to suffering from some congenital abnormalities. Occasionally, the cry recording process was performed with background noise or even with a constant noise from the care unit and from medical equipment that is connected to the infants who are in the NICU due to their prematurity or defects. Although the NICU should be a quiet environment for sleeping babies, in reality there is a large amount of unwanted noise from infusion pumps, monitors, ventilators, telephones and doors. Our goal is to develop a system that does not need complex and advanced technology to provide poor mothers having newborn babies in low-income countries. Thus, soundproof rooms or units were not used to record the cry signal to obtain the best signal-to-noise ratio that could otherwise be achieved. Each recorded infant's cry signal, even a healthy infant who is not completely clean, is manually segmented into 13 units or classes, which are defined and labeled in Table 1.

The case subjects for this study were infants selected from 1 to 53 days old, comprising healthy and sick full-term babies. For each infant, there are three recording files, with the average duration of 90 s for each continuous file. Useful information such as the date of birth were recorded along with the following pertinent information: weight, gender, maturity, race, ethnicity, gestational age, known and detected diseases, APGAR result, date and time of each cry recording and the reason for the crying (such as pain, hunger, diaper change, birth cry, medical exam). So far, we have divided the available health conditions in our cry database (CDB) into different categories listed in Table 2. The reason for the cry is not considered in the selected samples, in contrast to previous studies that used only the pain cry (Partanen et al., 1967).

## 3 Feature extraction

Usually, in human speech signals, there are low-level and high-level cues that can be used to recognize different speakers; these cues are related to the acoustic and semantic or linguistic aspects of speech. The human auditory system uses different levels of information, in contrast to automatic systems which depend still on low-level acoustic information. The major challenges of having a higher level of information derived from a cry signal are to find and extract some features in such a way that they convey distinctive information from the cry signal; this approach has been under study in recent years (Kheddache, 2014). In this paper, MFCCs are selected to be extracted as features in order to represent newborn infant cry signals since they have a good performance on various types of speech applications (Deller et al., 1993, Quatieri, 2002). Note that the same basic model of speech production (Deller et al., 1993) in adults is used to find these measurements. Thus far, it has been shown that they are also effective in classifying healthy and sick infants based on our primary results (Farsaie Alaie and Tadj, 2012). Moreover, we incorporate context information by adding dynamic features in this work, but they are not necessarily the most informative features for the intended pathology classification task.

### 3.1 Preprocessing stages

To increase the accuracy and reliability of the MFCC feature extraction process, cry signals are pre-processed in 3 simple steps:

#### 3.1.1 Convert stereo channel to mono channel

In the data collection step, because we have used a 2-channel recorder, we must average the channels first and then convert the signal into a single-channel signal using a mean value function.

#### 3.1.2 Pre-emphasization

Similar to in a speech recognition system, we used a first-order high-pass FIR filter to pre-emphasize the signal due to the high dynamic range of the digitized cry waveform, such as in a speech waveform. The main reason for using this filter is to compensate the spectral effect of the glottal source by introducing a zero near z=1 (Deller et al., 1993). Therefore, the filter $P\left(z\right)=1-0.97z^{-1}$ (Young et al., 2006, Rabiner and Schafer, 1978) should be applied prior to deriving the features or characteristics that correspond to the vocal tract only.

#### 3.1.3 EXP/INSV detection

Generally a cry is defined as the expiratory phase of respiration with sound or phonation by the larynx, which contains the vocal cords or folds and the glottis (LaGasse et al., 2005). Here, the input data (feature vector) given to our cry-based diagnostic system (cry pathology classifier) represents a processed version of one or more voiced expiration (EXP) or inspiration (INSV) segments of cry utterances. Therefore, after segmentation and labeling, only the EXP and INSV segments of the cry signals are selected for the feature extraction procedures. The system has been built manually by trained experts so far, but we are working on automatic segmentation of recorded cry signals that can act instead of voice activation detection (VAD) in speech recognition systems.

### 3.2 Static and dynamic MFCCs

**Fig. 2:**
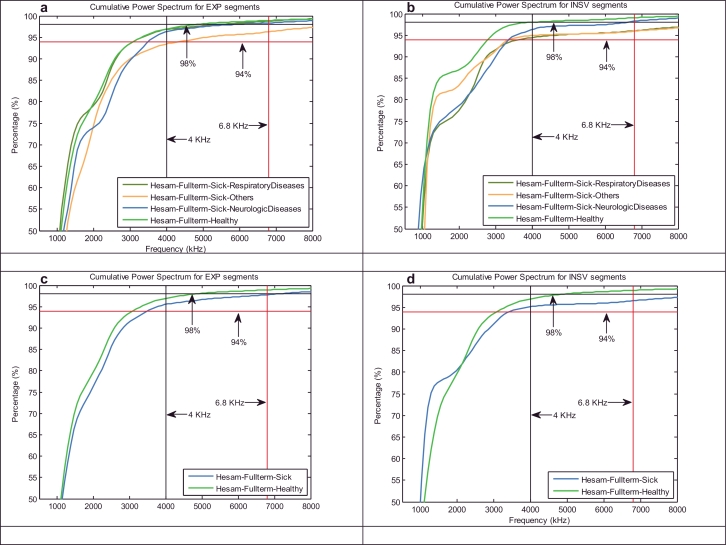
Cumulative power spectrum of (a) and (c) EXP units, (b) and (d) INSV units for each health condition.

**Fig. 3:**
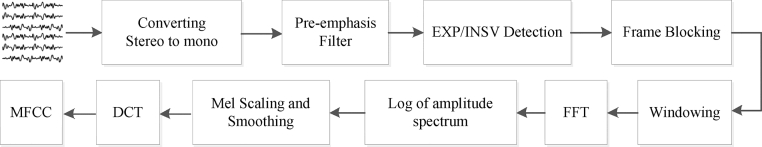
Pre-processing and MFCC feature extraction steps.

Briefly, the cry signals are pre-processed to be prepared for short-term processing, and then, the feature extraction procedure is applied. Generally, all of the conventional analysis techniques used in the signal processing application work with short-term frames of signals with non-stationary dynamics, such as human speech. Therefore, even in our case in point, it is our duty to select a reasonable portion of the cry signal in such a way that it does not change statistically. Frames are commonly 10–30 ms in duration, to be statistically stationary with a good tradeoff between the frequency and time resolutions in applications that use speech signals. In this paper, feature extraction was performed by using two different frame durations, 10 and 30, with the same overlap percentage (30%) between two consecutive windows, to find a good tradeoff between the frequency and time resolution of the cry signals and to assess what improvements it might have. After framing and prior to any frequency domain analysis, the hamming windowing has been applied to reduce any discontinuities at the edges of the selected region. In human speech signals, there is not much information above 6.8 kHz. The cumulative power spectrum is used here to detect the upper cutoff frequency of the efficient frequency band of infant cry signals, where the power almost stops to increase. The results of our experiments depicted in Fig. 2 demonstrate that the cry signals of full-term healthy and sick infants have almost 94% and 98% of their energies below 4 kHz and 6.8 kHz respectively. The results depicted in Fig. 2 also indicate that the energies of the inspiration segments for sick infants tend to accumulate at a slower rate than the energies of the expiration segments, especially in cases of RDS disorders. Thus far, information up to a 4 kHz bandwidth has been used, but we plan to conduct pioneering research on the aforementioned upper frequency band. In brief, the feature extraction phase for either the INSV or EXP-labeled segments can be performed in two stages:

1. Reduce the dimensionality by Cepstral analysis and extract the first 12 MFCCs computed from 24 filter banks plus the energy feature.
  MFCCs are introduced by Mermelstein in Davis and Mermelstein (1980) as the DCT of the log-energy output of the triangular bandpass filters. To extract the MFCCs, first the fast Fourier Transform (FFT) is performed to obtain the magnitude frequency of each windowed frame, and then, the MFCCs are calculated by converting the log Mel spectrum back to the time domain using the discrete cosine transform (DCT):
  (1) $$C_{i}=\sum_{k=1}^{K}S_{k}\cos\left[i\left(k-\frac{1}{2}\right)\frac{\pi }{K}\right],\;i=1,2,\ldots ,M$$
  where *K* is number of subbands (filter banks) (which is 24 for our selected bandwidth (Reynolds, 1995)), *M* is the desired length of the cepstrum, and *S_k_* represents the log-energy output of the *k*th triangular band pass filter. Fig. 3 indicates all of the 3 pre-processing steps followed by the aforementioned MFCC extraction procedure in stages.
2. Add dynamic features by taking the first and second derivatives of the obtained 13-static features, called the delta and delta–delta (acceleration) coefficients.

The first time derivation of the basic static parameters (referred to as delta coefficients) can be calculated over a limited window, as follows (Young et al., 2006):

(2) $$D_{n}=\frac{\sum_{\theta =1}^{\Theta }\theta \left(C_{n+\theta }-C_{n-\theta }\right)}{2\sum_{\theta =1}^{\Theta }\theta^{2}}$$

where *D* is a delta coefficient at the discrete time *n*, and *C_i_* shows the static parameters. Because the equation depends on both the past and future static parameters *C*_*n* ± *θ*_, to avoid having a problem with the regression window at the beginning and end of the static parameters, usually replication of the first and last parameters is required. The same formula is applied to the calculated delta coefficients to compute the second derivation of the static parameters (referred to as the acceleration coefficients). After appending the delta and acceleration coefficients to the static MFCC parameters, the set of 39-length feature vectors extracted from each single windowed frame cry is denoted *x_t_*, where *t* shows the sequence index. Therefore, a cry signal can be displayed by the sequence of feature vectors *x_t_* running up to the end of the signal, with *T* feature vectors $X=(x_{1},\ldots ,x_{t},...,x_{T})$.

## 4 Statistical modeling and descriptions

There is a minor difference between detection and identification systems in a decision process, while both use the same base of information. It has been shown that identification occurs inside of a detection task in some sense anyway, and their performances change together in the same way (Thomas, 1985). We are seeking to introduce a cry-based identification system to classify the presented infant as having one of the specified health conditions, but in this paper, detection is measured by the ability of our classifier to distinguish between an infant with the specified health condition and an infant with the other conditions listed in Table 2 as preliminary stages.

### 4.1 Likelihood ratio detector

In an ideal case with well-defined models for cry signals of infants in health and pathological conditions, the defined classification problem is similar to the canonical language recognition problem (Brummer, 2010) with the closed-set of specified languages. Similar to speaker identification systems that are intended for a 1:*N* match, the voice is compared against *N* speaker models $\left(\lambda_{1},\lambda_{2},\ldots ,\lambda_{N}\right)$, where *λ_i_* represents the parameters of the *i^th^i*th speaker model. This system can be presented by a maximum likelihood classifier whose objective is to select the speaker model that has the maximum a posteriori probability (MAP) for the observation vector sequence $X=(x_{1},\ldots ,x_{t},...,x_{T})$. The decision can be presented by the minimum-error Bayes’ rule, as follows:

(3) $$\begin{array}{ccc} \mathrm{Matched}\;\mathrm{Speaker}\;\mathrm{Index} & = & \arg\max_{1\leq i\leq N}\mathrm{Pr}\left(\lambda_{i}|X\right)\\ & = & \arg\max_{1\leq i\leq N}\frac{p\left(X|\lambda_{i}\right)\mathrm{Pr}\left(\lambda_{i}\right)}{p\left(X\right)} \end{array}$$

If it can be assumed that the prior probability of each speaker is equal, then the decision formula can be simplified as follows:

(4) $$\begin{array}{ccc} \mathrm{Matched}\;\mathrm{Speaker}\;\mathrm{Index} & = & \arg\max_{1\leq i\leq N}p\left(X|\lambda_{i}\right)\\ & = & \arg\max_{1\leq i\leq N}\sum_{t=1}^{T}\log p\left(x_{t}|\lambda_{i}\right) \end{array}$$

In verification systems, the task is a 1:1 match, which is in marked contrast to the identification system. For example, in speaker verification systems, the objective is to determine if the observed input *X* is from the hypothesized speaker (hypothesis *H*_0_) or not (hypothesis *H*_1_). The likelihood ratio detector has been accepted as a general approach in the speaker verification system (Reynolds et al., 2000). Assume that *H*_0_ and *H*_1_ hypotheses are represented by models *λ_Hyp_* and $\lambda_{\bar{Hyp}}$, respectively; it calculates the ratio of the posterior probabilities of the two hypotheses:

(5) $$\frac{Pr\left(\lambda_{Hyp}|X\right)}{Pr\left(\lambda_{\bar{Hyp}}|X\right)}$$

Bayes’ rule provides a shortcut for calculating the likelihood ratio in the log domain by ignoring constants that result in the log-likelihood ratios of *H*_0_ and *H*_1_, as follows:

(6) $$\Lambda \left(X\right)=\log p\left(X|\lambda_{Hyp}\right)-\log p\left(X|\lambda_{\bar{Hyp}}\right)\begin{array}{c} accept\;H_{0}\;\\ \underset{<}{\geq }\\ reject\;H_{0} \end{array}\theta$$

where *θ* is a threshold that adjusts the trade-off between two types of error, false acceptance and false rejection. Although the claimed speaker has a well-defined model in such a system, the corresponding alternative models are ill-defined. This issue poses a challenge to create $\lambda_{\bar{Hyp}}$ in such a way that presents the entire space of possible alternatives to the hypothesized speaker. In general, two main approaches have been described in Reynolds et al. (2000) to model $\lambda_{\bar{Hyp}}$. Since both techniques are evaluated in our experiments, we describe both of them briefly below.

(1) Background speaker models

In this approach, the set of speaker models excluding the hypothesized speaker have been selected and combined to model the alternative hypothesis. There has been a large amount of research into background speakers (Higgins et al., 1991, Matsui and Furui, 1994, Reynolds, 1995, Rosenberg et al., 1992). Given *B* equally likely background speakers, which are represented by $\left(\lambda_{1},\lambda_{2},\ldots ,\lambda_{B}\right)$, the log-likelihoods of the hypothesized speaker and alternative hypothesis (background speakers) are computed as (Reynolds, 1995)

(7) $$\log p\left(X|\lambda_{Hyp}\right)=\frac{1}{T}\sum_{t=1}^{T}\log p\left(x_{t}|\lambda_{Hyp}\right)$$

The 1/*T* factor is used to normalize the duration effect in the log-likelihood. Note that by ignoring the 1/*T* factor, the likelihood of the background speakers can be observed as the joint probability density of the observation *X* arising from one of the *B* background speakers:

(8) $$\log p\left(X|\lambda_{\bar{Hyp}}\right)=\log\left(\frac{1}{B}\sum_{b=1}^{B}p\left(X|\lambda_{b}\right)\right)$$

where *p*(*X*|*λ_b_*) is computed as in Eq. (7). The main drawback of this approach is preparing a background speaker set for each hypothesized speaker, which can be a problem for applications that have a large number of hypotheses.

(2) Speaker-independent model

This technique (Reynolds, 1997, Matsui and Furui, 1995) attempts to pool training samples from a large number of speakers, to represent the population of speakers by a single speaker-independent model; this model is currently known as a universal background model (UBM). A Universal Background Model (UBM) is a world model that is used mostly in biometric verification systems to represent general feature characteristics (Reynolds, 2009). Specifically, the universal-background model-based GMM or GMM-UBM has a large amount of success in statistical modeling techniques for speaker recognition and language recognition systems (Reynolds et al., 2000), and in contrast to previous approaches, a trained UBM can be used for all hypothesized speakers in the task.

### 4.2 Gaussian mixture models

The GMM modeling technique is simple but effective due to its remarkable ability to form smooth approximations from any arbitrarily shaped data distribution. It has been a success as a statistical model in different applications and systems, most notably in speaker recognition and speaker identification systems (Reynolds and Rose, 1995) due to its ability to model the underlying data classes or distributions of acoustic observations from a speaker. The likelihood function of a GMM used for a *D*-dimensional feature vector, *x*, is a weighted sum of *K* multivariate Gaussian components, *f_i_*(*x*), each parameterized by a *D* × 1 mean vector (*μ_i_*) and a *D* × *D* covariance matrix (*Σ_i_*), as given by the equation

(9) $$\begin{array}{ccc} F\left(x|\lambda_{K}\right) & = & \sum_{i=1}^{K}c_{i}f_{i}\left(x\right)=\sum_{i=1}^{K}c_{i}N\left(x|\Phi_{i}\right)\\ & = & \sum_{i=1}^{K}c_{i}N\left(x|\mu_{i},\Sigma_{i}\right) \end{array}$$

where *λ_K_* represents the GMM parameters and consists of *K* components with the restriction that the mixture weights must satisfy the following two constraints: *c_i_* ≥ 0 for i=1,…,K and $\sum_{i=1}^{K}c_{i}=1$. The *i*th component can be written in the following notation:

(10) $$\begin{array}{ccc} f_{i}\left(x\right) & = & N\left(x|\Phi_{i}\right)=N\left(x|\mu_{i},\Sigma_{i}\right)=\frac{1}{{\left(2\pi \right)}^{\frac{D}{2}}{\left|\Sigma_{i}\right|}^{\frac{1}{2}}}\\ & & \times \exp\left(-\frac{1}{2}{\left(x-\mu_{i}\right)}^{Tr}\Sigma_{i}^{-1}\left(x-\mu_{i}\right)\right) \end{array}$$

where $\Phi_{i}=\left(\mu_{i},\Sigma_{i}\right)$ are the parameters for the *i*th Gaussian density, and *A^Tr^* represents the transpose of matrix *A*. Collectively, a GMM can be denoted by its parameters as $\lambda_{K}=\left(c_{i},\Phi_{i},\;i=1,\ldots ,K\right)$.

### 4.3 System description

The proposed diagnostic system is built around the likelihood ratio test for verification, GMMs for likelihood functions and GMM-UBM models for adapting alternative health conditions via the adapted-BML method instead of common Bayesian adaptation (Reynolds et al., 2000, Duda and Hart, 1973, Gauvain and Chin-Hui, 1994). In the related literature, this approach is also known as Bayesian learning or the MAP estimation method. In our work, one UBM is a health-independent GMM that is trained with cry samples from the available training CDB that contains full-term healthy and sick infants with specific diseases, to represent the general cry feature characteristics. Another UBM is pathology-independent GMM that attempts to model cries from sick infants in available pathological classes. Then, we employed the adapted BML method to adapt the related UBM to a target or specific class. We will show that this approach improves the performance of our classifier in comparison to our reference system, which uses Bayesian adaptation.

The crux of the design is how we fuse subsystems into a single effective system. Our cry-based multi-class recognition system has a hierarchical scheme that is a treelike combination of individual classifiers in serial and parallel modes. We used the ability of the serial mode to narrow down the health condition of the infants to one of two possibilities, such as the biometric identification system introduced in Hong and Jain (1998). This approach means that in the first step, the two-class pattern recognizer should make the decision as to which proposition should be eliminated, healthy or sick infants. This part of our system acts as a verification system for healthy infants, while distinguishing between healthy infants and sick infants. The goal here is to determine whether the infant is healthy or not; in the case of unhealthy, the second part should act as an identification system because the cry signals of the sick infants are assumed to be from the predefined set of known sicknesses. The winner sickness best matches the test infant's cry signal model in a known group of diseases. This sickness identification system involves only the aforementioned enrolled sicknesses and not all of the newborn illnesses. In the closed-set case, $\left(N_{1},N_{2},\ldots ,N_{L}\right)$ represents L different infant sicknesses, which have well-defined statistical models. In the second scenario, which is called the open-set, the same $\left(N_{1},N_{2},\ldots ,N_{L-1}\right)$ are specified sicknesses, and *N_L_* denotes any of the unseen out-of-set sicknesses. Therefore, we created a class called “others” or “none-of-the-above” in the target set of diseases. The state-of-the-art infant's cry-based health care system has a hierarchical scheme that is composed of two subsystems that are both based on an acoustic approach. Individual scores for expiration (EXP)-based and inspiration (INSV)-based experts are fused together to exploit the complementary information that can be represented by our health care system defined over the features extracted from two different types of corpora.

For healthy infants, the decision process should be stopped at this stage before using all of the remaining classifiers that can reduce the overall recognition time. Then, in the case of sick infants, trained individual pathological detector systems, in a parallel mode of operation, should arrive at a final decision on the pathological condition of the test infant. In case the accuracy of the final decision on the most likely disease was called into doubt, there is another class called *others* that corresponds to infants that do not have the considered diseases or those for which we need more recorded cry signals for more examination.

The proposed detection system works in two phases, namely, training and runtime test. In the first phase, labeled cry corpora are analyzed and used to train the corresponding model. Each model should represent some health-dependent characteristics of the training data. In the test phase, the presented cry sample goes through the same process as in the training phase (the preprocessing and feature extraction steps), and then, for both the expiration and inspiration corpora of the sample, the log-likelihood ratio to the hypothesized model is calculated. In this paper, the performance of each EXP and INSV-based expert is evaluated and then, at the decision stage of the system, the obtained scores from these experts are fused to improve the performance.

### 4.4 Applying the GMM-UBM

In this paper, we defined and trained two GMM-UBMs for each corpora (EXP and INSV), as we mentioned earlier; the first one is a single health-independent background model trained to represent the distribution of the extracted cry features, regardless of what condition the infant might have (healthy or sick), and the second one is a pathology-independent background model. Because we focus our attention on full-term healthy and sick newborn infants that have specific diseases, we train the UBM to be used for the classification of healthy and sick infants using only corresponding data that are reflective of the expected alternative cry to be encountered during recognition. For example, in this case, it is known a priori that the cry signal belongs to a full-term infant, and thus, the full-term test infant will only be classified against full-term infant cries. This approach applies to both the gestational age of the infants and the types of diseases that are considered in our case.

Note that in this research project the recording process is still in progress. So far the collection and manual segmentation of data has been done at two different points in time. Thus we have two separate CDB called training and testing including completely different newborn infants. Table 3(a) displays information about training data (including available data for both training UBM and adaptation process at the time). The number of infants, the number of male and female, the number of usable cry signals in each class and total duration of expiration/inspiration segments in each class are shown. Note that the class named “Sick infants” contains all infants and their samples from the five pathology classes listed in Table 2. Moreover, the duration of usable EXP/INSV cry types in total were shown in Table 3(a) for each class. Our testing CDB will be described in detail later in Section 5.2 of this paper.

**Fig. 4:**
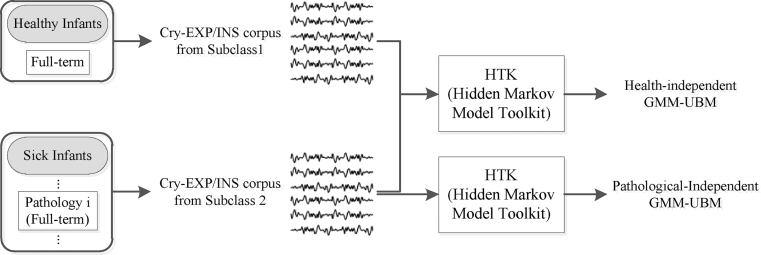
Balanced data pooling approaches for two defined GMM-UBM.

For both the EXP and INSV-based experts, the cry signals from subpopulations (healthy and sick infants with selected diseases) are pooled prior to training the health-independent UBM. Since we have more data samples in healthy infant class compare to sick class in total, there is a possibility of obtaining biased UBM toward the dominant subpopulation which is the healthy class. Therefore we exploit a portion of each subpopulation within the available training data (Table 3(a)) in such a way that we create a balanced training database over the subclasses for both predefined UBMs (Fig. 4) to avoid this problem. For this purpose, first we tried to select cry signals in such a way that, each pathology class (five pathology classes) has almost the same duration of EXP/INSV-labeled segments. Then, we selected healthy cry signals in a way that their inspiration and expiration durations are almost the same length as those of total cry signals in the sick class (including all 5 selected diseases).

Table 3(b) provides an overview of the number of infants and recorded cry signals, the duration of usable EXP/INSV cry type within the created balanced CDB for each healthy and pathological class. Note that the rest of training CDB which were not selected as a member of the created balanced CDB (Table 3(b)), were employed to derive the hypothesized models by adaptation of the UBMs. Similar to in speaker verification, there is no objective measure to determine the correct number of infants or the duration of cry signal to train a UBM. It is worthwhile recalling that the procedure for the data collection is still in progress; thus, we used all of the data that was available at the time for training each model and, then, the incoming data for the test and evaluation process.

Prior this work, we introduced the Adapted BML (Farsaie Alaie and Tadj, 2012) method to estimate mixture model parameters; this approach has better performance than the conventional EM-based re-estimation algorithm as a reference system for the GMM training step. The Adapted BML has several advantages over the mentioned reference system, but the distinct advantage is that it estimates the optimum number of components by iteratively adding new components in the direction that largely increases the predefined objective function. There is no guarantee that increasing the number of components in a GMM trained by HTK provides better system accuracy (Dobrovic et al., 2012), although the EM algorithm (Dempster et al., 1977) iteratively re-estimates the GMM parameters to monotonically increase the likelihood of the model for the vector of observations, in contrast to the adapted-BML, in which each new added component brings improvement in the predefined objective function. Despite this option, in the preliminary stages of our cry-based diagnostic system, the building or training phase of the defined GMM-UBMs has been performed based on the HTK (Hidden Markov Model Toolkit) software tool, which is an established tool of speech recognition systems based on hidden Markov models (Young et al., 2006). In the training procedure, we substitute diagonal covariance matrices for the full covariance matrices due to its computational efficiency because a diagonal covariance GMM with order *K* > 1 can model distributions of feature vectors with correlated elements. Then, in the next step, we used the adapted version of the parameter updating procedure described in Farsaie Alaie and Tadj (2012) to adapt UBM to create specific health condition models.

### 4.5 BML adaptation of sub-models or health-dependent-infant cry models

As mentioned earlier, there is a common technique called Bayesian adaptation (Duda and Hart, 1973, Gauvain and Chin-Hui, 1994) for deriving the hypothesized speaker model from GMM-UBM. Here, we introduce a new way of updating the GMM-UBM parameters based on the infant cry signals from related subclasses. In fact, a part of this adaptation technique was introduced earlier in Farsaie Alaie and Tadj (2012) and Jun et al. (2011) as partial and global updating in Boosted mixture learning (BML) of GMM and HMM-based acoustic models. Specifically, we use the concept of boosting to refine the UBM parameters using the training cry signals of infants with a specific health condition.

As mentioned earlier, the model parameters, $\lambda_{K}=\left(c_{i},\Phi_{i},\;i=1,\ldots ..,K\right)$, of each UBM with a known number of mixtures, *K*, can be calculated by using the HTK software tool. To adapt the obtained UBM, the statistics and sample weights, *W*(*x_t_*), of each subclass training data are calculated for each mixture, *f_k_*, in the UBM. Then, they are used to refine the corresponding mixture parameters, *Φ_k_*, and mixture weights, *c_k_*, iteratively, while $F_{K-1}$ are assumed to be constant. By applying the EM algorithm to optimize the log-likelihood of the model for the vector of observations only with respect to the mixture component *f_k_*, the iterative formula can be derived to adapt the model parameters. The adapted parameters,$\;{\hat{\lambda }}_{K}=\left({\hat{c}}_{i},{\hat{\Phi }}_{i},\;i=1,\ldots ..,K\right)$, can be estimated in the $\left(n+1\right)th^{\mathrm{th}}$ equation as follows:

(11) $$\begin{array}{ccc} w^{n}\left(X_{t}\right) & = & \frac{f_{k}\left(X_{t}|\Phi_{k}^{\left(n\right)}\right)}{c_{k}^{n}f_{k}\left(X_{t}|\Phi_{k}^{\left(n\right)}\right)+\left(1-c_{k}^{n}\right)F_{k-1}\left(X_{t}|\lambda_{k-1}\right)}\\ & = & \frac{f_{k}\left(X_{t}|\Phi_{k}^{\left(n\right)}\right)}{F_{k}\left(X_{t}|\lambda_{k}\right)}\\ \gamma_{t}\left(\Phi_{k}^{\left(n\right)}\right) & = & \frac{w^{n}\left(X_{t}\right)}{\sum_{t=1}^{T}w^{n}\left(X_{t}\right)}\\ {\hat{c}}_{k}^{n+1} & = & \frac{1}{T}\sum_{t=1}^{T}{\hat{c}}_{k}^{n}w^{n}\left(X_{t}\right)\\ {\hat{\mu }}_{k}^{n+1} & = & \sum_{t=1}^{T}\gamma_{t}\left(\Phi_{k}^{\left(n\right)}\right).X_{t}\\ {\hat{\Sigma }}_{k}^{n+1} & = & \sum_{t=1}^{T}\gamma_{t}\left(\Phi_{k}^{\left(n\right)}\right).\left(X_{t}-{{\hat{\mu }}_{k}}^{n+1}\right)\;{\left(X_{t}-{{\hat{\mu }}_{k}}^{n+1}\right)}^{Tr} \end{array}$$

in which the UBM parameters are used as an initial point. The adaptation procedure is performed in such a way that mixtures with a high count of subclass training data concentrate more on these examples, and vice versa. In other words, due to the existence of *f_k_* in the numerator and $F_{K-1}$ in the denominator of the weight samples equation, the observations that have lower probabilities by the $F_{K-1}$ model are given larger weights than those that have higher probabilities. It is worthwhile mentioning that the first part of the denominator can reduce the probability of the case in which *f_k_* is dominated by a few samples. Moreover, sample weights in the updating mixture weights formula act as a tuning parameter, which helps to rectify the mixture weights iteratively by determining the ability of each mixture component to model the subclass training samples.

In comparison to the clear coupling method presented in the Bayesian adaptation (Reynolds et al., 2000, Gauvain and Chin-Hui, 1994), the BML adaptation can be observed as an indirect or hidden coupling between both the mixture weights and the parameters of the adapted model and UBM. Note that in the Bayesian method, there are relevant factor and adaptation coefficients (Reynolds et al., 2000) that control the balance between the old and new estimates.

## 5 Evaluations and experiments

### 5.1 Defining GMM-UBM and adaptation methods

Specifically, we created two health-independent UBMs by training 875 and 92 mixture GMMs with pooled healthy and sick data, from the balanced database (Table 3(b)) for the EXP and INSV-labeled cry segments, called $\lambda_{HI-UBM-EXP}$ and $\lambda_{HI-UBM-INSV}$, respectively. Then, two pathology-independent UBMs that included 443 and 51 mixture GMMs were trained by only sick data for the EXP and INSV-labeled cry segments, called $\lambda_{PI-UBM-EXP}$ and $\lambda_{PI-UBM-INSV}$, respectively. We selected the number of mixtures based on the created UBM in the 1999 NIST SRE, which is a combination of 1024 mixture GMMs from using one hour of speech per gender (Reynolds et al., 2000). In the first classification task, healthy and sick models are derived from the health-independent UBMs, $\lambda_{HI-UBM}$, while in the pathology detection task two pathology models (infants with neurological and respiratory problems) are derived from the pathology-independent UBMs, $\lambda_{PI-UBM}$. Note that during adaptation of the UBMs, four different adaptation methods have been exploited in order to compare as follow:

1. MAP or Bayesian adaptation that adapts only the mean vectors – This approach has the best performance among all of the combinations of parameter adaptations for a speaker verification system (Reynolds et al., 2000). Moreover, it was mentioned that adapting the weights by MAP for known reasons degrades the overall performance.
2. BML adaptation method for refining the mean and variance vectors.
3. Coupling old and BML adaptation estimates over the mean and variance vectors – We compute new statistics for the parameters based on the BML model estimates, and we use a single adaptation coefficient for both the mean and variance parameters $\alpha_{i}=\frac{n_{i}}{n_{i}+r}$ with the relevant factor r=16 to control the balance, which is the same as in Bayesian adaptation.
4. BML adaptation method for refining only the mean vectors.

### 5.2 Log-likelihood scores computation

**Fig. 5:**
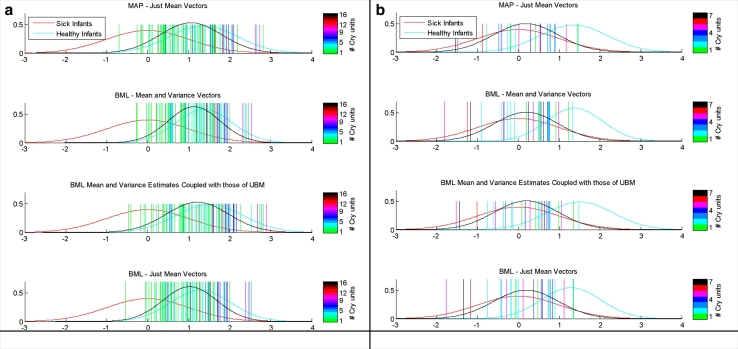
Mean of the LLR scores over INSV cry units inside the (a) healthy and (b) sick infants for the healthy infant verification system.

We applied the idea of the HNORM score normalization method described in Reynolds (1997) for the EXP/INSV-labeled cry segments separately. In each health-condition detector, we used only non-hypothesized or non-target cry samples (imposter) to estimate the normalization parameters. Therefore, the non-target log-likelihood ratio score distributions have been rescaled to have a mean of zero and a standard deviation of one. Due to different lengths of extracted EXP/INSV segments from each recorded cry signal, more evidence might be needed to make a reliable decision for each test file, especially for the inspiratory cry which has a shorter duration than that of the expiratory cry. Therefore, each corpus was split into small cry units of approximately 3 s duration to investigate the effect of the EXP/INSV duration length in each recorded file. The results indicate that independent of the frame length, type of cry units (EXP/INSV), adaptation method and task of the detector, recorded files that have more 3 s-length EXP/INSV cry units have more separable LLR scores (Fig. 5). In other words, the more information that is available (EXP/INSV length inside each file), the more likely the information leads to more reliable decision and less uncertainty about the detected pathological condition.

Earlier, we defined approximately 3-s duration of an EXP/INSV-labeled segment as an EXP/INSV cry unit. In general, cry signals include more expiratory cry segments than inspiratory cry segments, but the situation became worse because finding pure INSV segments was not likely. Table 4 displays the information about the testing CDB in which 89 and 101 cry signals of healthy and sick infant respectively were collected from new infants after the time of collecting training CDB (Table 3). The testing CDB described in Table 4 was collected just for evaluation and testing process. It is worthwhile mentioning that during the test process, in order to evaluate the effect of duration of available INSV/EXP-labeled segments in a test cry signal on the classification result, the entire of one recording file for a baby was considered as a test input not only one cry unit or a short segment of the test file. Thus, based on the available duration of EXP/INSV segments in samples, three different testing datasets can be defined from testing CDB (Table 4) for each cry type (EXP or INSV) as follows:

Test dataset A: Including cry signals with any length of INSV/EXP-labeled segments (Table 5(a))
Test dataset B: Including cry signals with at least one 3-s INSV/EXP cry unit (Table 5(b))
Test dataset C: Including cry signals with at least three 3-s INSV/EXP cry units (Table 5(c))

Table 5 shows the number of test cry samples more clear corresponding for the aforementioned test datasets. Moreover, it depicts a large reduction in the amount of both B and C test datasets after using two corresponding cry unit restrictions. In later sections of this paper we evaluated both EXP and INSV-based experts with all these three data bases but in some experiments only the best obtained results were presented.

### 5.3 Healthy-conditioned detector systems based on expiratory or inspiratory cry units

In this section, we present the results of both healthy and sick (with specific diseases) infant detection systems.

#### 5.3.1 Healthy infant detector

**Fig. 6:**
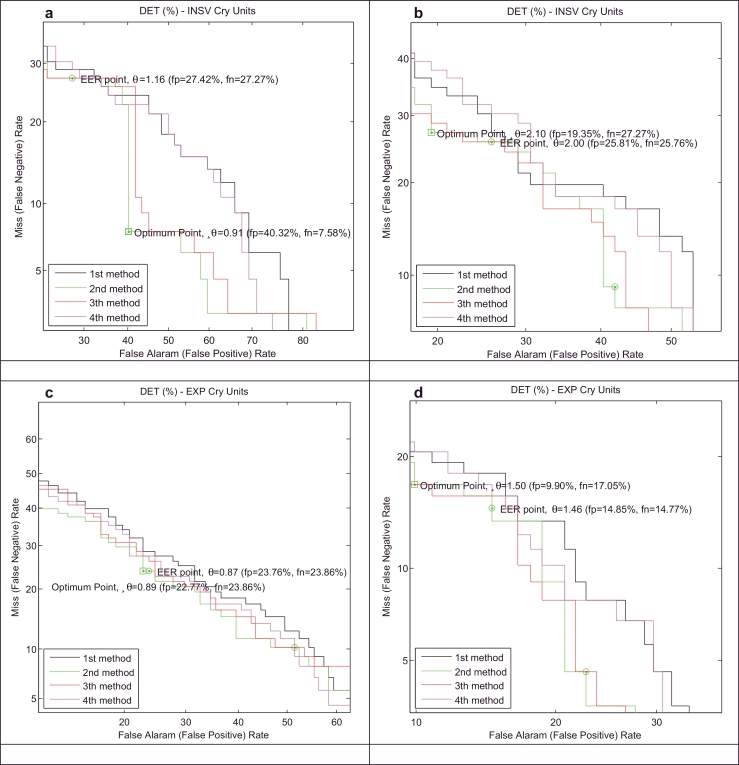
DET curves for two alternative hypothesized models, $\lambda_{PI-UBM}$ (a) and (c) and $\lambda_{\bar{Hyp}}$ (b) and (d), and for INSV (a) and (b) and EXP (c) and (d) cry units with a 10 ms frame length in the healthy infant verification system.

**Fig. 7:**
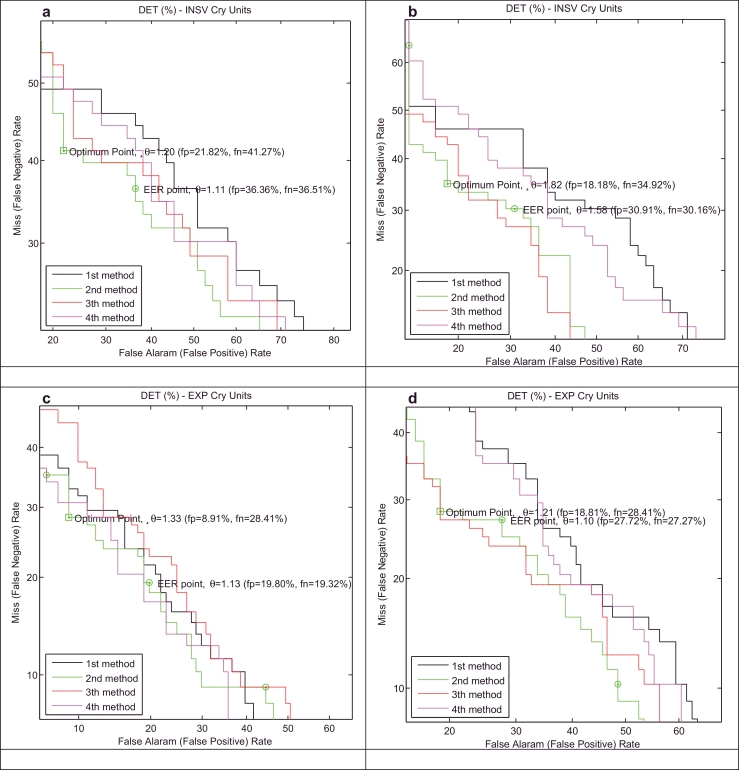
DET curves for the two alternative hypothesized models, $\lambda_{PI-UBM}$ (a) and (c) and $\lambda_{\bar{Hyp}}$ (b) and (d), and for INSV (a) and (b) and EXP (c) and (d) cry units with the 30 ms frame length in the healthy infant verification system.

We present the results of our healthy infant detector for a test database using both test EXP and INSV-labeled segments with two different frame lengths (10 ms and 30 ms). Moreover, to describe the entire space of possible alternatives for the healthy class, two explained approaches, called background speaker modes $\lambda_{\bar{Hyp}}=\left(\lambda_{1},\lambda_{2},\ldots ,\lambda_{B}\right)$ and UBM *λ*_UBM_, have been used to compute the LLR scores. Because some of the test data do not have 3-s INSV cry units, to evaluate the detector based on the INSV models, we performed our experiments on two sets of test datasets (Table 5): 1) containing INSV-labeled segments with any length (test dataset A) and 2) containing at least one 3-s INSV unit (test dataset B). On the other hand, almost all of the data from the test database contains pure EXP-labeled segments except for one sample, and thus, there are 88 healthy and 101 sick samples for the evaluation EXP-based expert. Here, we only present the results of test dataset B which are more satisfactory than those of test dataset A, as expected. The miss (false negative) and false alarm (false positive) rates are, respectively, plotted on the *x*- and *y*-axes, which are scaled non-linearly (normal deviate scale) as detection error tradeoff (DET) curves (Martin et al., 1997). The DET plots that are depicted in Figs. 6 and 7 distinguish more clearly the performance of the systems that have different adaptation methods (defined in Section 5.1), frame lengths, cry unit types and representatives for the alternative health conditions.

All of the points on the DET curves have different *FAR*(%) and *FPR*(%), and in practice, the operating point (OP) should be selected based on the task of the system in which all of the application criteria are met. For example, in biometric security systems, the point must have a low FAR. Because finding the best suitable OP in such a diagnostic system is not our concern in this paper, the equal error rate (EER) points are plotted by individual circle-shaped points on curves where FAR(t)=FPR(t),t∈S, and S is the set of thresholds for calculating the OP distribution. Note that an exact EER point might not exist. Moreover, the optimal ROC operating points described in Metz (1978) are shown by the square-shaped points on the curves. The decision threshold is selected in a way that minimizes the average cost at this point. The slope of the ROC at this point is given by

(12) $$S=\frac{C_{FP}-C_{TN}}{C_{FN}-C_{TP}}\times \frac{P\left(+D\right)}{P\left(-D\right)}$$

where *C_FP_*, *C_TN_, C_FN_, C_TP_* are the costs, and *P*(∓*D*) is equal to the probability that a case from the database is an ∓ case. Here, our predefined costs for computing *S* are as follows:

$$C_{FP}=C_{FN}=0.5,\;C_{TN}=C_{TP}=0$$

The overall accuracy and error rates depend on the chosen operating point, which is not clear here. Therefore, to compare the systems fairly and independently of the cutpoint, the Area under the ROC curve (AUC) is used as a measure of the performance of the each detector, while an ideal classifier has an AUC equal to 1. The value of EER (%) and AUC for the systems plotted in Figs. 6 and 7 are listed in Table 6. It is apparent that the experiments on the shorter frame length have better results in most of the cases and the EXP-labeled cry units have a more distinctive ability than the INSV-labeled cry units in classifying healthy and sick infants independent of the frame length, as we anticipated. Moreover, because in general each cry signal contains more expiratory cry units than inspiratory cry units, the average LLR score computed over EXP-labeled cry units is more reliable than the INSV-labeled cry units.

To show the impact of the number of cry units on the system performance, especially for the inspiratory cry, we used test dataset C (Table 5(c)) including only test files that have at least 3 cry units to perform the classification. Almost all of the cry test samples with EXP-labeled segments have more than 3 cry units (except for one healthy and two sick samples); therefore, the achieved results for the expiratory cry units are the same as the results in Table 6 again. However, this condition has a larger effect on the inspiratory segments of the recorded infant cry signals and reduces the number of test files to 32 and 23 for healthy and sick infants respectively. The results given in Table 7, which are independent of the frame length, adaptation method and background model, confirm that the more INSV-labeled cry units are inside a test file, the more chance at the end of the evaluation to diagnose it correctly.

Among the four adaptation methods defined earlier (Section 5.1), our reference system with the Bayesian or MAP adaptation method (method 1) has the lowest AUC, and the other specific methods, both 2-3, which use the BML adaptation estimates, have lower error-detection rates with a higher AUC. The system with the highest AUC for both the EXP and INSV-labeled cry segments is the system that uses the $\lambda_{\bar{Hyp}}$ background model and the 2nd method of adaptation (defined in Section 5.1). Therefore, the minimum achieved equal error rates are 14.85% and 25.8% for using the EXP and INSV-labeled cry segments, respectively.

#### 5.3.2 Sick infant detector with a specific disease

A lack of data, especially in the training data for a specific illness, causes difficulty in training and adapting well-defined models, such as the model for infants who have blood disorders. Even at the evaluation time, there is not a sufficient number of test samples in all of the diseases (Table 4). Thus far, we have only used our detector for sick infants who suffer from neurological and respiratory disorders. In the interest of brevity, only the results for a shorter frame length (10 ms) and background speaker models $\lambda_{\bar{\mathrm{Hyp}}}$ over test dataset B, which are more satisfactory than other results, will be discussed.

There are limited numbers of distinct sick infants in training CDB (Table 3) who are not the same as the infants collected in the testing CDB (Table 4). In total, 10 and 5 infants are used to train the respiratory and neurological disease models, respectively. In comparison to previous results in the healthy infant detector system, very low training errors plus test results depicted in Table 8 for the unseen test dataset B (Table 5(b)) might be a sign of memorizing training data rather than learning. This finding is due to an apparent lack of enough distinct infants in each corresponding class, especially for neurological disease. It is important to understand that the data collection process, training and adapting procedures are time-consuming, but that also, in spite of them, further corresponding full-term sick infants (as with healthy infants) result in better generalization by training well-defined models. Even using cross-validation, which is a method for preventing overfitting, is not a quick-fix solution. Therefore, collecting new data to increase the size of the training CDB to rebuild a pathology-independent background model and sickness models is a practical solution to improving the performance.

It has been shown in Table 8 that among adaption methods (defined in Section 5.1) those which use BML technique (the 2nd, 3rd and 4th methods) have the highest AUC than our reference system with the Bayesian adaptation. Moreover, according to the result in Table 8, it is clear that EXP and INSV cry unit-based models have different ability to distinguish available classes. For example, INSV model has better performance than EXP model in Respiratory disorder detector system, but on the other hand in neurological disorder detector system, EXP model has better performance than INSV model. Therefore, we tried to take advantage of the strength of each classifier (EXP and INSV cry unit-based models) as a separate source of information, known as decision fusion.

### 5.4 Fusion, calibration and decision

A more sophisticated system can be developed by integrating the evidence presented by multiple sources of information, similar to in multimodal biometric systems. Such a multimodal system is expected to be more reliable in contrast to a unimodal system, which relies on the evidence of a single source. Generally speaking, the strategy of fusion can be categorized into three levels, which are called the data or feature level, matching score level, and decision level (Blum and Liu, 2005). Although the feature set is richer in discriminative information than the matching score or the output decision of a classifier, fusion at the match score level is usually preferred because it is relatively easy to obtain and there is no need to worry about the feature compatibility at the score level or rigid fusion at the decision level (Ross and Jain, 2004). Here, the fusion of the two proposed subsystems (expiratory and inspiratory cry unit-based GMM) can be done by integration at matching score level since obtained LLR scores (the output of EXP and INSV-cry unit based models) are the quality of each match.

There are two different strategies for consolidating scores obtained from different classifiers. The first approach formulates it as a combination problem. The final decision is made by a single scalar score which is a combination of matching/individual scores (Ben-Yacoub et al., 1999, Dieckmann et al., 1997). Note that before combination, the scores must be first transformed to a common domain. There are several techniques for addressing the combination problem like the sum rule, median rule, product rule, min/max/median rules and majority voting (Li et al., 2013, Snelick et al., 2005). The second approach treats it as a classification problem and constructs a feature vector using the matching scores output by the individual classifiers/matchers (Verlinde and Cholet, 1999, Vatsa et al., 2007). The obtained feature vector is then classified into one of two “Accept” or “Reject” classes. In contrast to first approach, there is no need of preprocessing to have homogeneous individual scores.

In this paper, the classification approach has been used to information fusion at matching score level. So we considered matching scores at the output of EXP and INSV-cry unit based models as a two-dimensional feature vector. In other word, the LLR scores obtained from two individual classifiers (EXP/INSV cry unit-based model) are concatenated to construct a two-dimensional feature space. Several classifiers have been used to consolidate the obtained individual scores and arrive at a decision: multilayer perceptron (MLP) using the back-propagation algorithm, probabilistic neural networks (PNN) and a support vector machine (SVM) (Table 9). These classifiers were evaluated using three test datasets (Table 5) that included cry signals containing both EXP and INSV labeled segments.

### 5.5 Results and discussion

To compare the generalizability of these three different algorithms and to find out the best algorithm for the available data, stratified K-fold cross-validation is used in which each fold has a roughly equal size and contains the same percentage of samples of each target class as in the whole dataset. Although 10-fold cross-validation is more common, in practice, usually the choice of the number of folds depends on the size of the dataset. Although there are different variants of cross-validated estimates (Refaeilzadeh et al., 2009), stratified 10-fold cross-validation is recommended by Kohavi (1995) as the best model. To obtain reliable performance estimation, multiple rounds of cross-validation are performed to test new and different random splits that result in smaller variance in the results and reduce the variability. Then, the validation results with three different values of *K*, depicted in Table 10, are averaged over the corresponding number of rounds.

The performances of the classifiers that use different adaptation methods (defined in Section 5.1) are compared based on some widely used statistical measures, namely, the false positive rate, false negative rate, accuracy, sensitivity and specificity. These statistical measures can be calculated from the classifier's results, as described in Fawcett (2006).

The error of the classifier follows a binomial distribution with the following mean and standard deviation:

$$\mu_{error}=p_{cv},\;\sigma_{error}=\sqrt{\frac{p_{cv}\left(1-p_{cv}\right)}{n}}$$

where *p_cv_* is the mean of *K* errors, and *n* is the number of samples. We can approximate the 100(1−α)% confidence interval for the error by the Wald confidence interval (Agresti and Coull, 1998), as follows:

$$p_{cv}\mp z_{\alpha /2}\sigma_{error}$$

where *z_β_* is the 1−β quantile of the standard normal distribution.

**Fig. 8:**
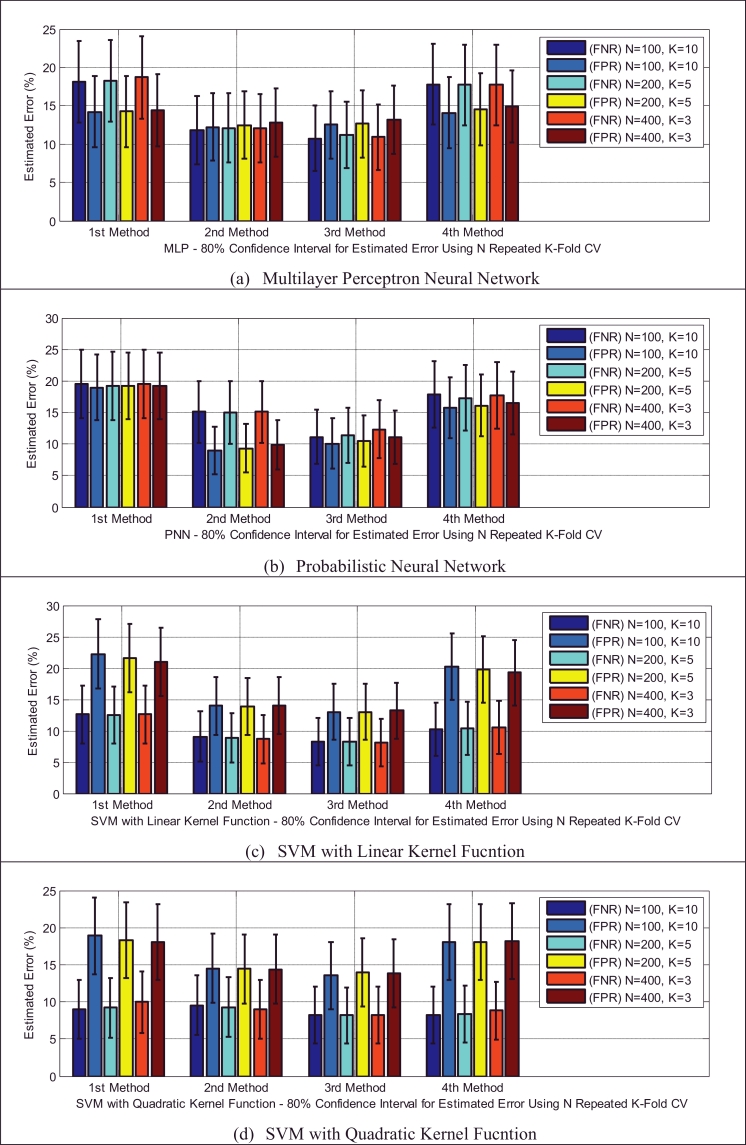

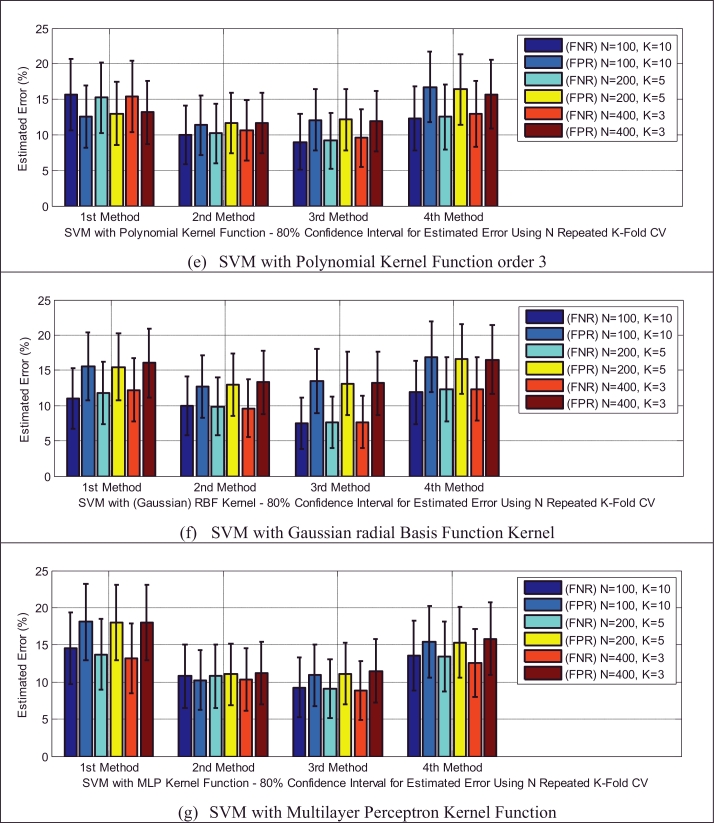
Type I and type II errors of the tested different healthy infant detector systems for each of the adaptation methods.

Based on Table 5, the test dataset A consists of 86 and 93 cry samples of healthy and sick infants whose recorded cry signals contain both EXP and INSV-labeled segments with any duration. These data are used to detect healthy infants by fusion of the obtained likelihood ratio scores from their expiratory and inspiratory cry units. Fig. 8 indicates both types of errors, the false positive rate (FPR or type I error) and the false negative rate (FNR or type II error), with an 80% confidence interval after applying stratified K-fold cross-validation for each classifier. Due to space limitations, we only plot the test error which is the average error that results from using a statistical learning method to predict the response on an observation in the test fold, one that was not used in training phase of K-fold cross validation. For each method of adaptation (defined in Section 5.1), there is not much difference in the errors between the K-fold cross-validations with different values of folds. The Bayesian adaptation method (1st method), which is our reference method, has obviously higher errors than the other methods, especially the 2nd and 3rd methods. To obtain a better comparison between the performances of the adaptation methods (defined in Section 5.1), Table 11 depicts the average accuracy, sensitivity and specificity rates over all of the used classifiers for each adaptation method. Here, the accuracy rate indicates the proportion of true classified infants (both healthy and sick) among the total number of infants in the test dataset A (Table 5(a)). The sensitivity measures the portion of healthy infants that are correctly identified, while the specificity measures the proportion of sick infants that are correctly verified. As is clear, the BML adaptation method for refining both the mean and variance vectors (2nd method) and the coupled BML adaptation estimations with old estimations (3rd method) are superior to the others, and even the BML adaptation for refining only the mean vectors (4th method) performs better than the Bayesian adaptation method (1st method).

**Fig. 9:**
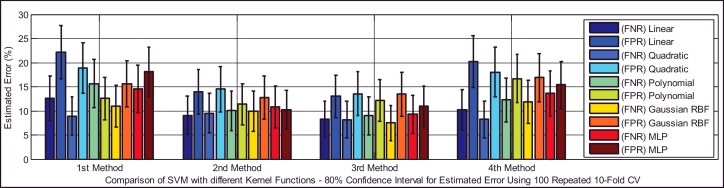
Type I and type II errors for the SVM with different kernel functions in the healthy infant detection task.

**Fig. 10:**
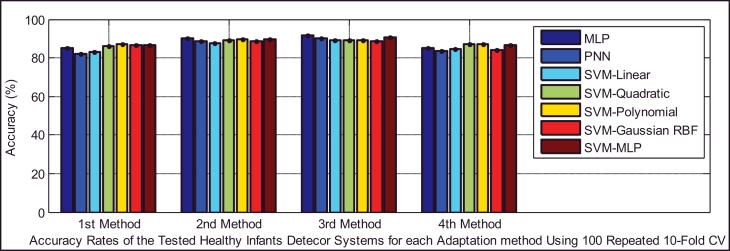
Comparison of the accuracy rates of all of the classifiers in the healthy infant detection task.

Classifiers provide different false negative and false positive rates, but as we expected from the fusion approach, even in the worst case of each classifier using the 2nd and 3rd adaptation methods, we obtained a smaller error rate than the lowest equal error rate of the model using EXP and INSV-labeled segments separately, which are 14.85% and 25.8%, respectively. The type I error and type II error were decreased to 8.84% for the false negative rate and 11.49% for the false positive rate using SVM-MLP with the 3rd adaptation method. The system with a smaller false positive leads to more false negatives, and vice versa. In biometric systems, the system with an approximately equal false rejection rate and false acceptance rate is called a tuned system (Tipton and Krause, 2007), and the goal is to tune this system to obtain an equal error rate that is as low as possible. Among all of the used classifiers, based on the error rates and accuracy rates depicted in Fig. 8, Fig. 9, Fig. 10, MLP and SVM with the MLP kernel function perform better than the others by providing almost the same type I and II errors (close to the EER point). Table 12, Table 13, Table 14 indicate in detail the accuracy rate, sensitivity and specificity of the classifiers as statistical measures of the performance of the classification test.

**Fig. 11:**
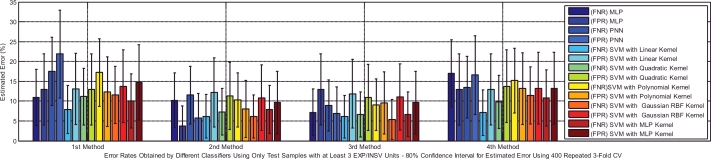
Type I and type II errors of the classifiers used in the healthy infant detection task, using 400 repeated 3-fold CVs for the test samples that contain more than 3 EXP and INSV-labeled cry units.

Earlier, we showed that using test samples with more INSV cry units can reduce the equal error rate down to 13% (Table 6). Likewise, in the next experiment, we used only those cry samples that had more than 3 cry units (test dataset C – Table 5(c)). We imposed this condition on existing samples in the test folds and due to the small resulting test data, 3-fold cross-validation is applied to evaluate the performance of the classifiers. The error reduction is clear by comparing the obtained results depicted in Fig. 11 with individual results for each classifier on the entire set of test dataset depicted in Fig. 8. For example, in the PNN classifier using the 3rd adaptation method, the type I error and type II errors were decreased to 6.8% and 8.8%, respectively.

After the first step of our cry-based diagnostic system, if the test infant is identified as a sick infant who suffers from one of our available diseases listed in Table 2, we should test it again with an available sick infant detector system to identify the most probable sickness. It is worthwhile mentioning that because of having a small number of infants and cry samples in our CDB at this time, only two types of diseases (neurological and respiratory diseases) were considered for sick infant detector system.

**Fig. 12:**
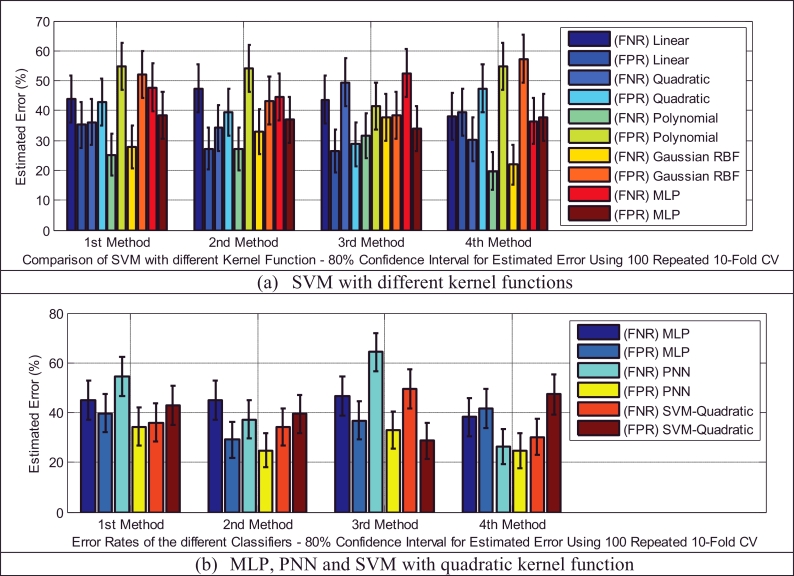
Type I and type II errors for the different classifiers in the sick infant (affected by neurological problems) detection task.

**Fig. 13:**
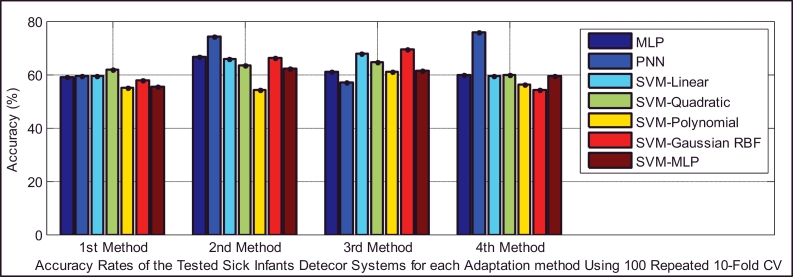
Comparison of the accuracy rates of all of the classifiers in the sick infant (affected by neurological problems) detection task.

In the test dataset A (Table 5(a)), we have 29 and 45 cry samples recorded from sick infants who suffer from neurological and respiratory disorders, respectively. For the first task, we had an imbalanced dataset (29 target and 64 non-target cry samples), and there are a few ways to address this problem; these approaches can be divided into data and algorithmic levels (Han et al., 2005). Because the dataset was small, the idea of under-sampling or data reduction techniques that remove only a majority of class samples is not an ideal solution. Another approach is to apply a higher misclassification cost to the minority class. This approach can balance out our imbalanced dataset, but here we assumed equal costs for the two types of error. Consequently, we simply increased the size of our minority class (target class) by duplicating the data. Although it was reported in Kolcz et al. (2003) that usually duplicating samples in a dataset has a detrimental effect on the model and accuracy rate, many re-sampling methods (Han et al., 2005) have been proposed in data mining algorithms as a solution to imbalanced data sets. Here, simple random over-sampling has been performed to balance the data set through duplicating some random samples of the minority class. It was observed that smaller error rates resulted for the created balanced data set. Fig. 12 indicates the type I and type II errors of the classifiers in the sick infant (those affected by neurological problems) detection task for the balanced data set. For the sake of brevity, we have illustrated only the error rates of the classifier using 100 repeated 10-fold cross-validation in these two sick infant detection tasks. As depicted in Fig. 13, almost all of the classifiers in this task that used either the 2nd or 3rd adaptation methods (defined in Section 5.1) have higher accuracy rates, except for PNN using the 4th adaptation method, in contrast to other adaptation methods. According to Table 15, it appears that the 3rd method is more focused on specificity, while the 4th method is more focused on sensitivity. In this case, the sensitivity and specificity contribute to the overall accuracy by having different weights, which is not ideal due to the equal cost assumption. Moreover, Table 15 indicates that the 2nd method has almost equal and the highest proportions of either actual healthy or sick infants that are correctly identified, among the adaptation methods.

**Fig. 14:**
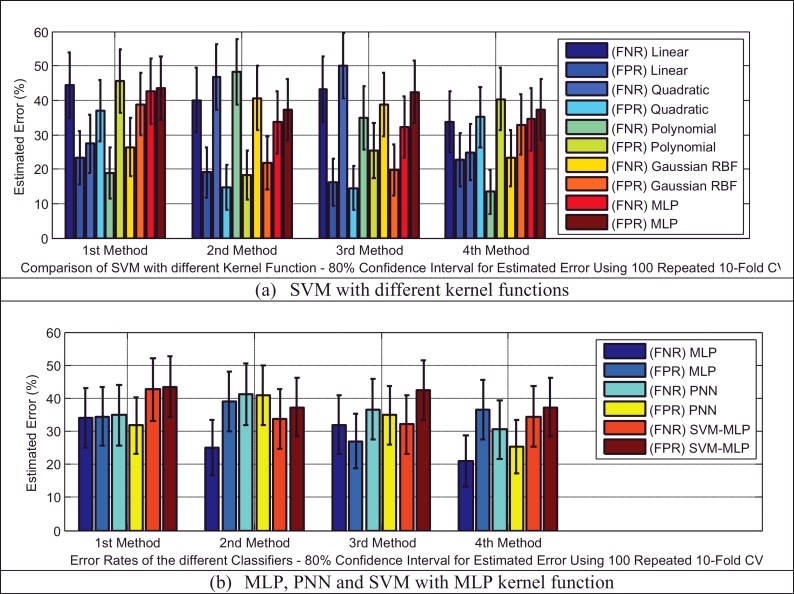
Type I and type II errors for the different classifiers in the sick infant (affected by respiratory disorders) detection task.

**Fig. 15:**
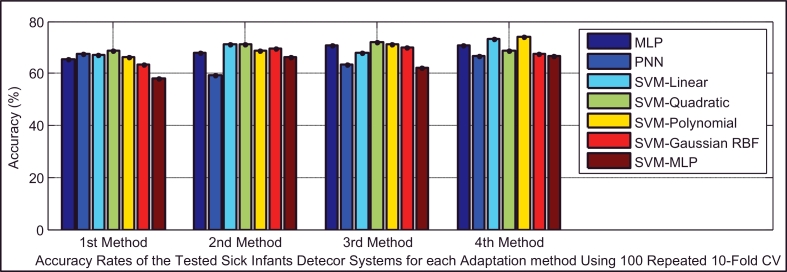
Comparison of the accuracy rates of all of the classifiers in the sick infant (affected by respiratory disorders) detection task.

Because the respiratory disorders class has more infants and also more cry samples than the neurological problems class (Tables 4 and 5), on average, the sick infant (affected by respiratory disorders) detection task performed slightly better, with a smaller generalization error than that for neurological disorders. The type I and type II errors of the classifiers are depicted in Fig. 14. It appears that adapting only the mean vectors by the BML method (the 4th method) has smaller error rates for all of the classifiers and the PNN classifier has more balanced error rates than the others (30.5% false negative, 25.4% false positive error rates). The statistical measures of the performance of the test are summarized in Table 16, which indicates that the 4th adaptation method is more accurate than the others (Fig. 15), while it is more focused on specificity (the same as the first method), in contrast to the other two methods, which are more focused on sensitivity.

## 6 Conclusions and further discussion

In this paper, we used the potential of newborn infant cry signals, which indicate the integrity of the central nervous system, to introduce a cry-based diagnostic system. The principal aim of the proposed system is to broaden the diagnostic system to address the most life-threatening illnesses and defects that occur in newborn infants. We believe that by developing this system we will create new possibilities for infants who suffer from birth defects or undetectable diseases, to obtain the treatment faster and protect them from health threats. In contrast to previous studies on infant cry signals, which usually address binary classification tasks, a hierarchical scheme that consists of subsystems is proposed to narrow down the health condition of an infant to one of the possibilities that is considered in our experiments; most of these possibilities have not been previously studied. It is worthwhile mentioning that due to the motivation for developing such a system in low-income countries without complex and advanced technology, there is no constraint placed on the recording steps of the cry signals. The fact that our cry data set is not clean and noise-free could harm the analysis process and reduce the NCDS system performance. After the analysis of infant cry vocalizations and the manual segmentation, the dynamic MFCC features along with static MFCC features are selected and extracted for both type of expiratory and inspiratory cry units to make a discriminative feature vector.

Here, in our infant health-condition verification/detection system, the UBM is an infant-independent GMM that is trained with cry samples from our cry dataset based on the HTK (Hidden Markov Model Toolkit) software tool to represent the general acoustic characteristics of crying. Then, for both healthy and sick infants with enrolled diseases, a unique cry-pattern has been trained by the adaptation procedure. In our previous papers (Farsaie Alaie and Tadj, 2012, Alaie and Tadj, 2013), we showed that the BML method, compared to other learning mixture models, has the great advantage of adding new mixture components in such a way that the greatest improvement is obtained in the predefined objective function. We used this idea to present a novel adapted BML process to derive subclass models from the GMM-UBM. The Bayesian adaptation method is assumed here to be our reference system for the adaptation procedure. The paper compares the obtained results for Bayesian and three different variants of adapted BML methods on each classification task.

Both types of expiratory (EXP) and inspiratory (INSV) cry units have their own ability to classify test infants for each task. Therefore, a score level fusion of the proposed two subsystems is performed to make a more reliable decision. In brief, our methodology has two main sequential steps, which are called encoding and making a decision. In the encoding stage, we have shown that how a cry signal represents itself in both the expiration and inspiration cry units within the GMM models by a set of extracted MFCC feature vectors and how to create log-likelihood ratio scores. In the next step, the decision on each test infant's cry was based on whether the set of feature vectors observed in the presented cry signal was more likely to have been produced by the hypothesized health-condition class or by the other available alternative classes. The fusion of the obtained individual scores was performed to construct a new feature vector.  The efficiency of the various classifiers, such as SVM, MLP, and PNN, had been evaluated to compare the adaptation methods (defined in Section 5.1) in each task.

Two health-independent and pathology-independent GMMs were trained by pooling the corresponding training data in a balanced version of our training CDB together and we used the remainder for adapting the subclass models. Then, testing was performed on the testing CDB with unseen infants, to evaluate the accuracy of each model. The results indicated that independent of the type of the cry units (EXP/INSV), adaptation method and task of the detector, the experiments on a shorter frame length (10 ms) have lower equal error rates (EER) than the 30 ms frame length in most of the cases. Moreover, based on the obtained results, the Bayesian or MAP adaptation method (1st method) not only has a higher EER but also has a lower AUC than the other three variants of the adapted BML method. In the first task, which was designed to detect healthy infants from sick infants who suffer from specific diseases according to the optimal likelihood ratio test, 14.85% and 25.8% equal error rates were achieved for the EXP and INSV-labeled cry units, respectively. This finding indicates that there is a higher ability for expiratory cry to distinguish healthy and sick full-term infants in contrast to a sick infant detector for respiratory disorders, while the trained model based on inspiratory cry has a lower error rate than those based on expiratory cry.

Although the presence of noise in the database has caused a loss of some portion of the INSV and EXP sounds in the baby cry audio recordings, the fact that the duration of the expiratory sounds is usually larger than the inspiratory sounds in newborn infant cries makes the average LLR score computed over expiratory cry units a more reliable estimate of the true value than the inspiratory cry units. We have shown that the length of the expiratory or inspiratory sounds that are available in a test file has a direct effect on the classification performance. In other words, the more information that is available (the EXP/INSV length inside each file), the more likely it is to have a more reliable decision and less uncertainty about the detected pathological condition. Those infants whose sample cries do not contain sufficient evidence should be asked to provide more samples. We concluded from the results on the healthy infant and sick infant (with neurological and respiratory disorders) that the ability of each cry type can vary by the type of disease or the general health condition.

Afterward, the obtained scores for each cry sample were used to construct a feature vector to combine expiratory and inspiratory cry-based individual scores. Several classifiers have been employed to evaluate the performance of the methods of adapting healthy and pathological subclasses at this stage. The error rates dropped from $\mathrm{EE}R_{\mathrm{EXP}}=14.85\%\;\mathrm{and}\;\mathrm{EE}R_{\mathrm{INSV}}=25.8\%\;$in the healthy infant detector systems to 8.84% false negative rate and 11.49% false positive rate using SVM-MLP with a variant of the BML adaptation method. Likewise, the error rates in sick infant (with neurological and respiratory disorders) detector systems were decreased by using score level fusion, and they even reached a 26.4% false negative and 24.6% false positive error rate and a 30.5% false negative and 25.4% false positive error rate using the PNN classifier with a variant of the BML adaptation method (4th method) on each task, respectively. Overall, the 2nd and 3rd adaptation methods, which use both the mean and variance vectors of the BML adaptation estimates, have almost the highest accuracy, sensitivity and specificity among the adaptation methods. The poor performance of the specified disease models (especially for infants with neurological disorders) in comparison to healthy and sick infant models is largely due to having a limited number of infants. In other words, the corresponding cry recordings did not contain enough variability to train GMMs in a way that obtained model represents all the possible observations.

While this paper has studied GMM-UBM approach in the area of infant cry classification, there are several open areas where research can improve from the introduced approach. As our future work, instead of relying on low-level acoustic features, we are considering the use of other cry features, such as percentage of either Hyperphonation or Dysphonation, and trying to find the most distinctive features among them for specific disease to improve the classification results. Then, multiple classifier combination, as each classifier is an expert in some different feature space, can be considered to combine their outputs. Meanwhile we are growing our CDB to contain enough variability in a sufficient amount to represent each considered health condition.

## Figures and Tables

**Table 1:** Different units available in the CDB.

Labels	Definitions
EXP	Voiced expiration segment during a period of cry
EXPN	Unvoiced expiration segment during a period of cry
INS	Unvoiced inspiration segment during a period of cry
INSV	Voiced inspiration segment during a period of cry
EXP2	Voiced expiration segment during a period of pseudo-cry
INS2	Voiced inspiration segment during a period of pseudo-cry
PSEUDOCRY	Any sound generated by the baby and it is not a cry
Speech	Sound of the nurse or parents talking around
Background	Kind of noise so low, it is characterized by a very low power-silence affected with little noise.
Noisy cry	Any sound heard with the cry e.g. machine's bip, water, diaper etc….
Noisy pseudo-cry	Any sound heard with the pseudo-cry
Noise	Like the sound caused by the mic moved by someone, the diaper, a door sound, speech + background, speech + bip.
BIP	sound of the medical instruments next the baby

**Table 2:** List of health-conditions.

Categories	Description
Healthy infants	Full-term infants without any major disorder or sickness
Heart problems	Full-term infants suffering from tetralogy of fallot, thrombus, complex cardio or congenital heart diseases
Neurological disorders	Full-term infants suffering from sepsis or meningitis
Respiratory diseases	Full-term infants suffering from respiratory distress or asphyxia diseases
Blood abnormalities	Full-term infants suffering from hyperbilirubinemia or hypoglycemia diseases
Other	Full-term infants suffering from other abnormalities or physical problems which are not in priority order for our system

**Table 3:** Number and duration of the recorded cry signals that were available in the training CDB at the time.

Class	Number of infants	Number of cry signals in training CDB	Overall length of training CDB	
			INSV	EXP
Healthy infants	58 (4 male)	142 (11 male)	12’4’’	92’3’’
Sick infants	25	66	3’53’’	41’25’’
Heart	4 (1 male)	12 (3 male)	34’’	5’4’’
Neurological	5 (3 male)	11 (7 male)	51’’	8’2’’
Respiratory	10 (5 male)	27 (15 male)	1’08’’	17’
Blood	3 (2 male)	9 (5 male)	36’’	5’06’’
Others	3 (no male)	7 (no male)	43’’	4’5’’
(a) Training cry database (CDB)				
Class	Number of infants	Number of cry signals in balanced training CDB	Overall length of balanced CDB	
			INSV	EXP
Healthy infants	39	53	2’40’’	25’25’’
Sick infants	22	54	3’03’’	26’06’’
Heart	4	12	34’’	5’40’’
Neurological	5	9	34’’	5’30’’
Respiratory	7	17	35’’	4’49’’
Blood	3	9	36’’	5’06’’
Others	3	7	43’’	4’50’’
(b) Training balanced cry database				

**Table 4:** Number of infants and recorded cry signals available in the testing CDB at the time.

Class	Number of infants	Number of cry signals in testing CDB
Healthy infants	42 (4 male)	89 (11 male)
Sick infants	40	101
Heart	2 (2 male)	3 (3 male)
Neurological	11 (6 male)	30 (18 male)
Respiratory	18 (12 male)	49 (35 male)
Blood	4 (4 male)	9 (9 male)
Others	4 (2 male)	10 (4 male)

**Table 5:** Number of cry samples in defined three different testing data sets from testing CDB available in Table 4.

	Healthy cry signals	Sick cry signals	Heart	Neuro	Resp	Blood	Others
INSV model	86	93	1	29	45	9	9
EXP model	88	101	3	30	49	9	10
(a) Test dataset A							
	Healthy cry signals	Sick cry signals	Heart	Neuro	Resp	Blood	Others
INSV model	66	62	1	22	24	7	8
EXP model	88	101	3	30	49	9	10
(b) Test dataset B							
	Healthy cry signals	Sick cry signals					
INSV model	32	23					
EXP model	87	99					
(c) Test dataset C							

**Table 6:** Comparison of the different healthy infant detector systems based on the equal error rate and area under the curve for all of the test samples.

INSV-$\lambda_{HI-UBM}$	10 ms		30 ms	
	Equal error rate (%)	AUC	Equal error rate (%)	AUC
'method1′	29.03	0.77	41.81	0.62
'method2′	27.41	0.815	36.36	0.69
'method3′	27.41	0.811	38.18	0.68
'method4′	29.03	0.78	40	0.64
INSV-$\lambda_{\bar{Hyp}}$	Equal error rate (%)		Equal error rate (%)	AUC
'method1′	27.41	0.806	38.18	0.68
'method2′	25.80	0.8350	30.90	0.77
'method3′	25.80	0.8355	29.09	0.81
'method4′	29.03	0.80	34.54	0.71
EXP-$\lambda_{HI-UBM}$	Equal error rate (%)	AUC	Equal error rate (%)	AUC
'method1′	27.72	0.81	20.79	0.87
'method2′	23.76	0.84	19.80	0.89
'method3′	24.75	0.826	22.77	0.86
'method4′	25.74	0.827	18.81	0.89
EXP-$\lambda_{\bar{Hyp}}$	Equal error rate (%)	AUC	Equal error rate (%)	AUC
'method1′	15.8415	0.932	32.67	0.76
'method2′	14.8514	0.951	27.72	0.825
'method3′	15.8415	0.951	24.75	0.824
'method4′	15.8415	0.932	30.69	0.77

**Table 7:** Comparison of the different healthy infant detector systems based on the EER and AUC for the test samples that have more than 3 INSV units (32 and 23 cry samples of healthy and sick infants respectively).

	10 ms		30 ms	
INSV-$\lambda_{HI-UBM}$	Equal error rate (%)	AUC	Equal error rate (%)	AUC
'method1′	13.043	0.933	25	0.75
'method2′	13.043	0.944	30	0.79
'method3′	13.043	0.941	35	0.77
'method4′	13.043	0.938	25	0.78
INSV-$\lambda_{\bar{Hyp}}$	Equal error rate (%)	AUC	Equal error rate (%)	AUC
'method1′	26.08	0.872	25	0.81
'method2′	17.39	0.888	25	0.82
'method3′	21.73	0.887	25	0.85
'method4′	26.08	0.877	20	0.82

**Table 8:** Results of the sick infant detector systems for respiratory and neurological disorders.

	Respiratory diseases			
	INSV-$\lambda_{\bar{Hyp}}$		EXP-$\lambda_{\bar{Hyp}}$	
10 ms	Equal error rate (%)	AUC	Equal error rate (%)	AUC
'method1′	28.94	0.80	34.61	0.75
'method2′	23.68	0.82	32.69	0.74
'method3′	26.31	0.81	36.53	0.73
'method4′	21.05	0.84	30.76	0.77
	Neurological disorders			
10 ms	Equal error rate (%)	AUC	Equal error rate (%)	AUC

'method1′	40	0.607	33.80	0.727
'method2′	47.5	0.587	28.16	0.777
'method3′	47.5	0.572	29.57	0.770
'method4′	42.5	0.631	30.98	0.746

**Table 9:** Training parameters used in SVM, PNN and MLP.

PNN	Number of neurons in layer 1 : 161
	First layer transfer function : Radial basis transfer function
	Spread value: 0.1
	Number of neurons in layer 2 : 2
	Second layer transfer function : Competitive transfer function
	Performance function : mse
	Learning algorithm : Scaled conjugate gradient
MLP	Number of hidden layers : 1
	Hidden layer neurons : 10
	Hidden activation function : Hyperbolic tangent sigmoid
	Output layer neurons : 2
	Output activation function : Softmax (normalized exponential)
	Max number of iteration : 1000
	Performance function : Crossentropy
	Learning algorithm : Scaled conjugate gradient
SVM	Number of iterations : 15,000
	Kernel functions :
	1- Linear
	2- Quadratic
	3- Polynomial (order 3)
	4- Gaussian RBF Kernel
	5- Multilayer perceptron Kernel with SMO method to find the separating hyperplane

**Table 10:** Number of folds and rounds used in the experiments.

Value of *K*	3	5	10
Number of iterations	400	200	100

**Table 11:** Average accuracy, sensitivity and specificity over the used classifiers in the healthy infant detection task.

	Method 1	Method 2	Method 3	Method 4
Average accuracy	85.21	89.13	89.76	85.98
Average sensitivity	88.07	89.85	90.73	88.64
Average specificity	82.53	88.37	88.41	82.74

**Table 12:** Accuracy rates for the used classifiers in the healthy infant detection task.

Classifier	Method 1	Method 2	Method 3	Method 4
MLP	85.03	89.95	91.68	84.99
PNN	82.11	88.79	89.93	83.82
SVM-linear	83.12	87.67	89.33	84.82
SVM-quadratic	86.11	88.98	88.98	87.25
SVM-polynomial	86.99	89.82	89.27	87.08
SVM-Gaussian RBF	86.59	88.78	88.75	84.33
SVM-MLP	86.57	89.85	90.41	86.60
Average accuracy	85.21	89.13	89.76	85.98

**Table 13:** Sensitivity rates for the used classifiers in the healthy infant detection task.

Classifier	Method 1	Method 2	Method 3	Method 4
MLP	84.86	90.83	93.05	91.11
PNN	82.77	87.08	89.44	83.88
SVM-linear	89.58	89.44	91.80	90.55
SVM-quadratic	93.19	90.97	92.08	93.19
SVM-polynomial	85.69	90.27	90.27	88.19
SVM-Gaussian RBF	88.47	89.72	90.83	86.25
SVM-MLP	91.94	90.69	90.69	87.36
Average sensitivity	88.07	89.85	90.73	88.64

**Table 14:** Specificity rates for the used classifiers in the healthy infant detection task.

Classifier	Method 1	Method 2	Method 3	Method 4
MLP	85	89	90.22	79.77
PNN	81.66	90.22	90.22	83.77
SVM-linear	77.11	85.88	87	79.33
SVM-quadratic	79.55	87.11	86.11	81.66
SVM-polynomial	88	89.22	88.11	85.88
SVM-Gaussian RBF	84.88	88.11	87	82.77
SVM-MLP	81.55	89.11	90.22	86
Average specificity	82.53	88.37	88.41	82.74

**Table 15:** Average accuracy, sensitivity and specificity over the used classifiers in the sick infant (affected by neurological problems) detection task.

Neurological	Method 1	Method 2	Method 3	Method 4
Average accuracy	58.26	64.57	63.15	60.67
Average sensitivity	57.85	63.3	54.76	67.85
Average specificity	58.29	65.06	66.25	57.03

**Table 16:** Average accuracy, sensitivity and specificity over the used classifiers in the sick infant (affected by respiratory disorders) detection task.

RDS	Method 1	Method 2	Method 3	Method 4
Average accuracy	65.22	67.68	68.18	69.59
Average sensitivity	68	61.85	61.71	74
Average specificity	62.85	73.50	74.50	65.71
